# Nitrous Oxide Production in Sputum from Cystic Fibrosis Patients with Chronic *Pseudomonas aeruginosa* Lung Infection

**DOI:** 10.1371/journal.pone.0084353

**Published:** 2014-01-17

**Authors:** Mette Kolpen, Michael Kühl, Thomas Bjarnsholt, Claus Moser, Christine Rønne Hansen, Lars Liengaard, Arsalan Kharazmi, Tanja Pressler, Niels Høiby, Peter Østrup Jensen

**Affiliations:** 1 Department of Clinical Microbiology, Rigshospitalet, Copenhagen, Denmark; 2 Department of International Health, Immunology and Microbiology, Faculty of Health Sciences University of Copenhagen, Copenhagen, Denmark; 3 Marine Biological Section, Department of Biology, University of Copenhagen, Helsingør, Denmark; 4 Plant Functional Biology and Climate Change Cluster, University of Technology Sydney, Sydney, Australia; 5 Singapore Centre on Environmental Life Sciences Engineering, Nanyang Technological University, Singapore, Singapore; 6 Copenhagen CF Centre, Rigshospitalet, Copenhagen, Denmark; University of Duisburg-Essen, Germany

## Abstract

Chronic lung infection by *Pseudomonas aeruginosa* is the major severe complication in cystic fibrosis (CF) patients, where *P. aeruginosa* persists and grows in biofilms in the endobronchial mucus under hypoxic conditions. Numerous polymorphonuclear leukocytes (PMNs) surround the biofilms and create local anoxia by consuming the majority of O_2_ for production of reactive oxygen species (ROS). We hypothesized that *P. aeruginosa* acquires energy for growth in anaerobic endobronchial mucus by denitrification, which can be demonstrated by production of nitrous oxide (N_2_O), an intermediate in the denitrification pathway. We measured N_2_O and O_2_ with electrochemical microsensors in 8 freshly expectorated sputum samples from 7 CF patients with chronic *P. aeruginosa* infection. The concentrations of NO_3_^−^ and NO_2_^−^ in sputum were estimated by the Griess reagent. We found a maximum median concentration of 41.8 µM N_2_O (range 1.4–157.9 µM N_2_O). The concentration of N_2_O in the sputum was higher below the oxygenated layers. In 4 samples the N_2_O concentration increased during the initial 6 h of measurements before decreasing for approximately 6 h. Concomitantly, the concentration of NO_3_^−^ decreased in sputum during 24 hours of incubation. We demonstrate for the first time production of N_2_O in clinical material from infected human airways indicating pathogenic metabolism based on denitrification. Therefore, *P. aeruginosa* may acquire energy for growth by denitrification in anoxic endobronchial mucus in CF patients. Such ability for anaerobic growth may be a hitherto ignored key aspect of chronic *P. aeruginosa* infections that can inform new strategies for treatment and prevention.

## Introduction

Cystic fibrosis (CF) is an autosomal recessive disease. It is caused by mutations in the cystic fibrosis trans-membrane conductance regulator gene [1] affecting apical ion transport. In the lungs, the defective ion transport results in endobronchial accumulation of thick, viscous mucus that prevents mucociliar cleaning of the lungs, and increases susceptibility to chronic respiratory infections [2], [3]. *Pseudomonas aeruginosa* is a Gram-negative, gamma proteobacterium, which dominates chronic lung infections in CF patients and is considered the most serious complication of CF [4], [5]. The chronic *P. aeruginosa* lung infection in CF patients is characterized by presence of endobronchial biofilm aggregates surrounded by numerous polymorphonuclear leukocytes (PMNs) [6], [7]. Despite the bactericidal activity of the PMNs and intensive antibiotic therapy, these biofilms persist and grow in the endobronchial mucus of CF patients over many years [7], [8]. *P. aeruginosa* can withstand the bactericidal activity of the PMNs by forming biofilms of the protective mucoid phenotype [9] and by quorum sensing (QS)-regulated production of leukolytic amounts of rhamnolipid [10]–[13]. The summoned PMNs produce reactive oxygen species (ROS) through a respiratory burst, which leads to intense depletion of molecular oxygen (O_2_) [14], a common feature of infected endobronchial mucus in CF [6]. Biofilm formation may explain why *P. aeruginosa* survives the attacking PMNs, but it is not known how *P. aeruginosa* acquires the energy required for the observed growth in endobronchial secretions [8] when O_2_ is absent. However, *P. aeruginosa* can grow anaerobically with alternative electron acceptors or by arginine fermentation [15], and it has been suggested that *P. aeruginosa* can respire by denitrification in anoxic CF mucus utilizing nitrate (NO_3_^−^) and nitrite (NO_2_^−^), which are both present in sufficient amounts [15], [16]. Although the ability of *P. aeruginosa* to utilize reduction of NOx for anaerobic respiration is well known [17], denitrification in mucus and persistent biofilms present in the airways of CF patients remains to be demonstrated. Since N_2_O is a natural intermediate belonging to the gases defining denitrification [17], we used electrochemical microsensors [18] to measure O_2_ and N_2_O concentration gradients at high spatio-temporal resolution in freshly expectorated sputum from CF patients with chronic *P. aeruginosa* lung infection.

Further evidence for denitrification was obtained from nitrate (NO_3_^−^) and nitrite (NO_2_^−^) turnover measurements in the sputum samples. These measurements provided important new insights to the micro-environmental conditions and chemical dynamics associated with persistent *P. aeruginosa* lung infections in CF patients and indicate that nitrogen compounds can play an important role in the interaction between pathogenic bacteria and an active immune response.

## Results

### N_2_O and O_2_ in sputum from CF patients with chronic *P. aeruginosa* lung infection

**Figure 1:**
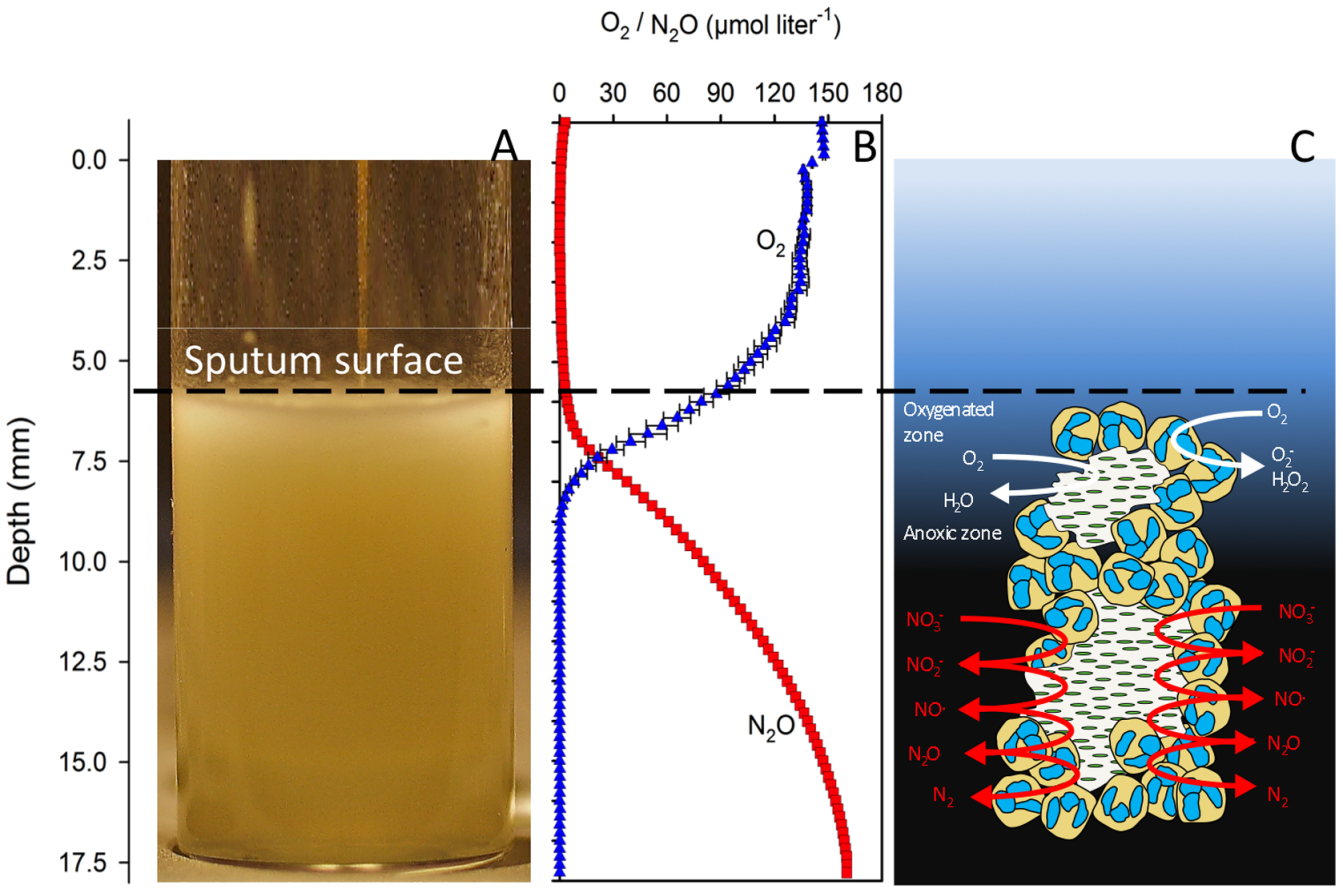
Microsensor measurements of chemical gradients in sputum. (A) Close up of a sputum sample from a cystic fibrosis patient with chronic *P. aeruginosa* lung infection with an inserted microsensor. (B) Representative microprofiles of N_2_O and O_2_ in a CF sputum sample. O_2_ profiles are shown as the mean and SD of three microprofiles recorded in the beginning of the experiment and did not change significantly throughout the experimental period, while the N_2_O profile represents the maximal N_2_O levels measured about 6–7 h after beginning. (C) A schematic model of the involved PMN and biofilm processes in CF sputum explaining the microprofiles.

Representative measurements of O_2_ and N_2_O in freshly expectorated sputum were acquired with O_2_- and N_2_O microsensors (Fig 1A). Measurements of O_2_- and N_2_O profiles in expectorated sputum from a CF patient with chronic *P. aeruginosa* lung infection showed the distribution of an upper oxygenated zone and a lower anoxic zone. The N_2_O profile reached the maximal concentration of N_2_O in the lower anoxic part of the sputum sample, suggesting that denitrification is mainly confined to the anoxic zone. A slow decline of O_2_ was apparently detected above the sputum surface. This may be because the position of the sputum surface was estimated by visual inspection, which is associated with uncertainty due to small amounts of heterogeneous saliva (Fig 1B).

Sputum is composed of heterogeneously distributed bacterial aggregates surrounded by PMNs consuming O_2_, and this respiratory burst creates local anoxic microenvironments in the sputum [14]. The metabolic mechanisms are thus compartmentalized according to the availability of O_2_ with an oxygenated zone, wherein the majority of O_2_ is reduced to superoxide by the summoned PMNs, and an anoxic zone, where *P. aeruginosa* can utilize nitrate as electron acceptor during oxidative phosphorylation (Fig. 1C).

### NO_3_^−^ and NO_2_^−^ in sputum from CF patients with chronic *P. aeruginosa* lung infection

**Figure 2:**
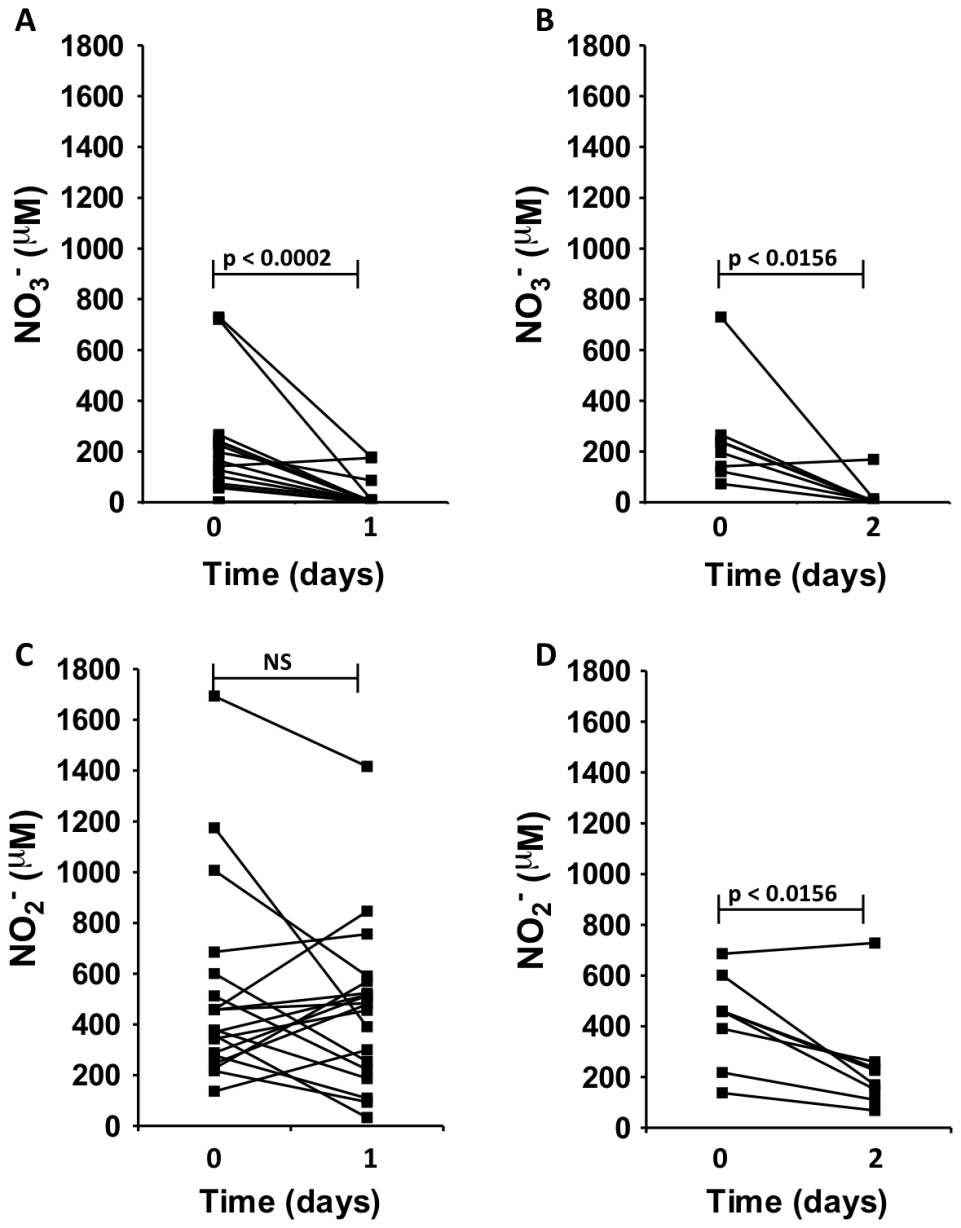
Consumption of NO3^−^ and NO2^−^ in sputum. (A, B) NO_3_^−^ concentration in sputum samples from cystic fibrosis patients with chronic *P. aeruginosa* lung infection. (C, D) NO_2_^−^ concentration in sputum samples from cystic fibrosis patients with chronic *P. aeruginosa* lung infection. Samples were collected immediately after expectoration and after 1 (n = 20) and 2 days (n = 7) of incubation. Data were analyzed by Wilcoxon signed rank test.

NO_3_^−^ and NO_2_^−^ concentrations in sputum samples were measured before N_2_O profiling and 1 day later (Fig 2). The concentration of NO_3_^−^ was significantly higher immediately before N_2_O profiling as compared to 1 and 2 days after incubation indicating NO_3_^−^ depletion due to ongoing denitrification (Fig 2A, B). The NO_2_^−^ concentration was not changed significantly after one day (Fig 2C), but by including additional measurements of the NO_2_^−^ concentration in 7 sputum samples a significantly decreased NO_2_^−^ concentration was detected (Fig 2D).

### Distribution of N_2_O in sputum from CF patients with chronic *P. aeruginosa* lung infection

**Figure 3:**
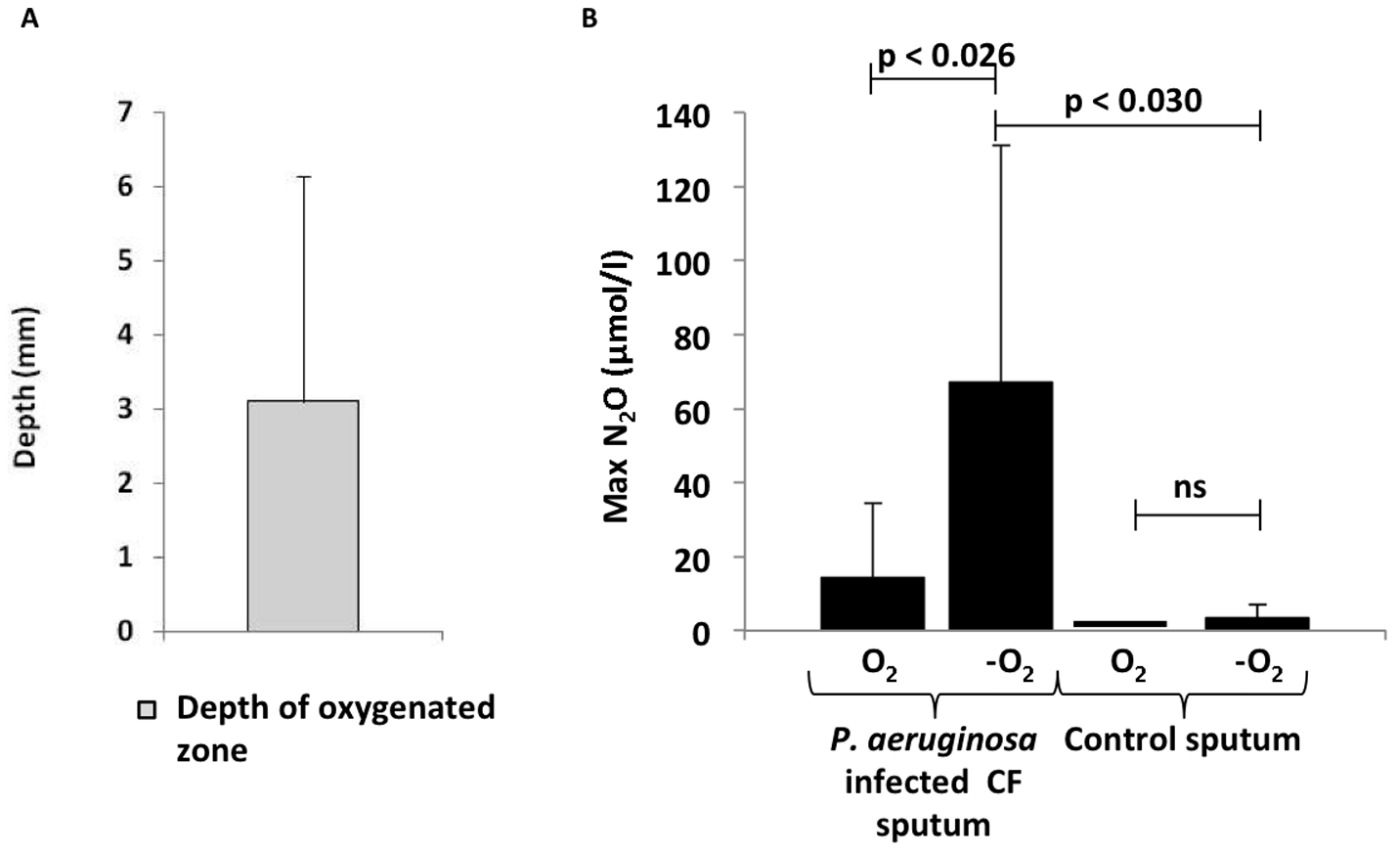
Distribution of O_2_ and N_2_O in sputum. (A) Depth of oxygenated zone in sputum samples from cystic fibrosis patients with chronic *P. eruginosa* lung infection (n = 8). (B) Maximal N_2_O concentration in the oxic zone and anoxic zone in sputum samples from cystic fibrosis patients with chronic *P. aeruginosa* lung infection (n = 8). Control sputum without detectable *P. aeruginosa* was obtained from two PCD patients and one CF patient. Statistical analysis was performed by Student t-test.

Vertical profiles of O_2_ in sputum samples showed depletion of O_2_, indicating the formation of anoxic zones below a mean depth of 3.1 mm (SD  = 3.0 mm) from the sputum surface (Fig 3A) suggesting that the average depth of O_2_ penetration of ∼3 mm. A higher concentration of N_2_O was observed in the anoxic zone as compared to the oxic zone (p<0.026, n = 8) (Fig 3B). To verify that N_2_O is related to *P. aeruginosa* we found significantly less N_2_O in three control sputum samples from 1 CF patient and from 2 primary ciliary dyskinesia (PCD) patients without detectable *P. aeruginosa* (p<0.030).

### Dynamics of N_2_O in sputum from a CF patient with chronic *P. aeruginosa* lung infection

**Figure 4:**
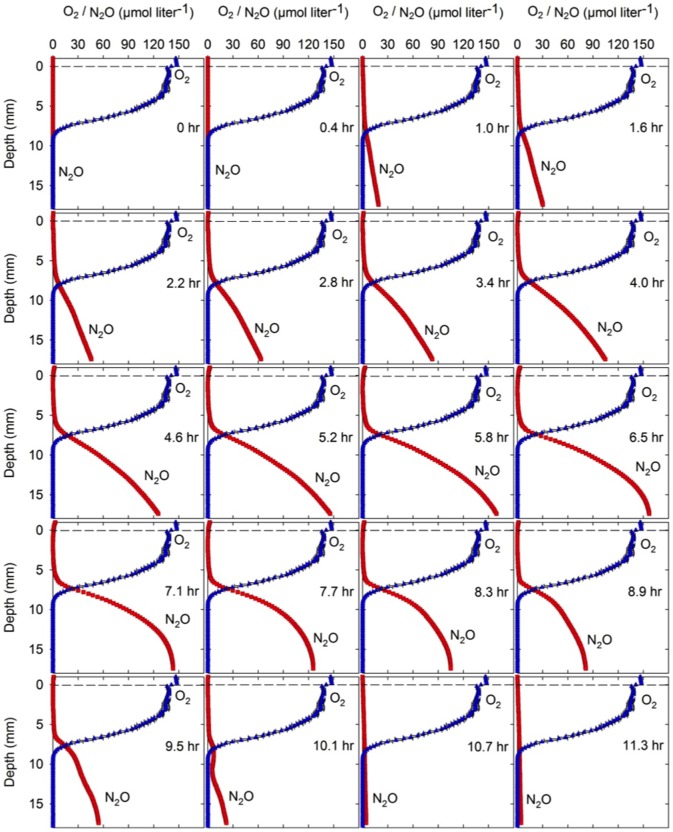
Generation and depletion of N_2_O in sputum. Spatio-temporal dynamics of N_2_O concentration profiles in a representative sputum sample from a cystic fibrosis patient with chronic *P. aeruginosa* lung infection showing initial accumulation of N_2_O in the anoxic zone followed by total depletion. The O_2_ concentration profile is shown as the mean and SD of three microprofiles recorded at the beginning of the experiment.

Figure 4 displays time series of representative N_2_O profiles measured vertically through a sputum sample. The distribution of O_2_ is displayed at 0 hr. During the initial measuring period, N_2_O accumulated in the anoxic zone reaching a maximum concentration of 160 µM after 6.5 h incubation, which indicates ongoing production of N_2_O. Within the subsequent 4 hours the accumulated N_2_O decreased indicating consumption through reduction to N_2_.

### Rates of N_2_O production and consumption in sputum samples

**Figure 5:**
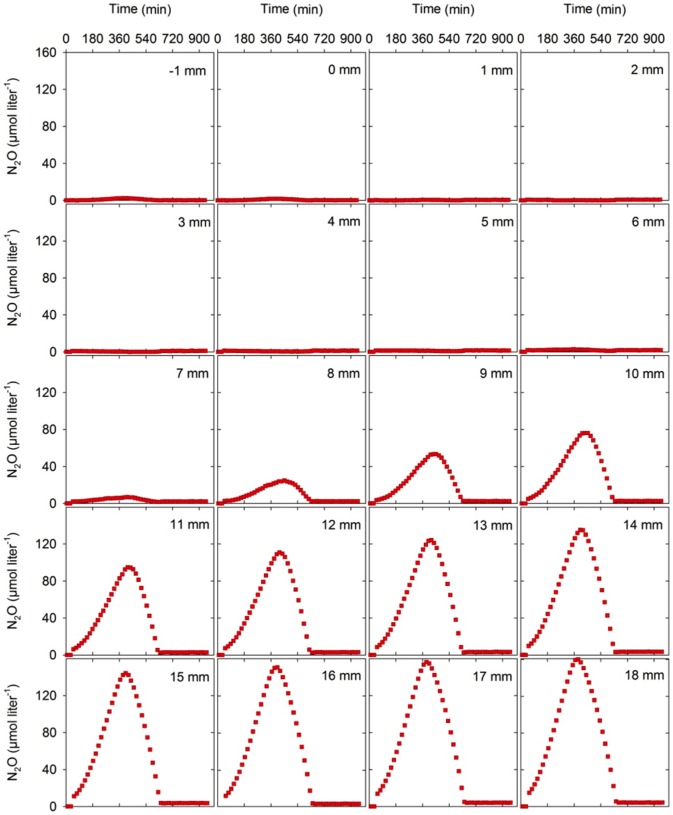
Rates of N2O production and consumption in sputum. Depth specific plots of N_2_O concentration vs. time at particular measuring depths in the same sputum sample as displayed in Fig 4. Accumulation and thus net production of N_2_O in all depths was observed until approximately 6 h, followed by net consumption of N_2_O presumably due to depletion of nitrate around 6 h.

**Figure 6:**
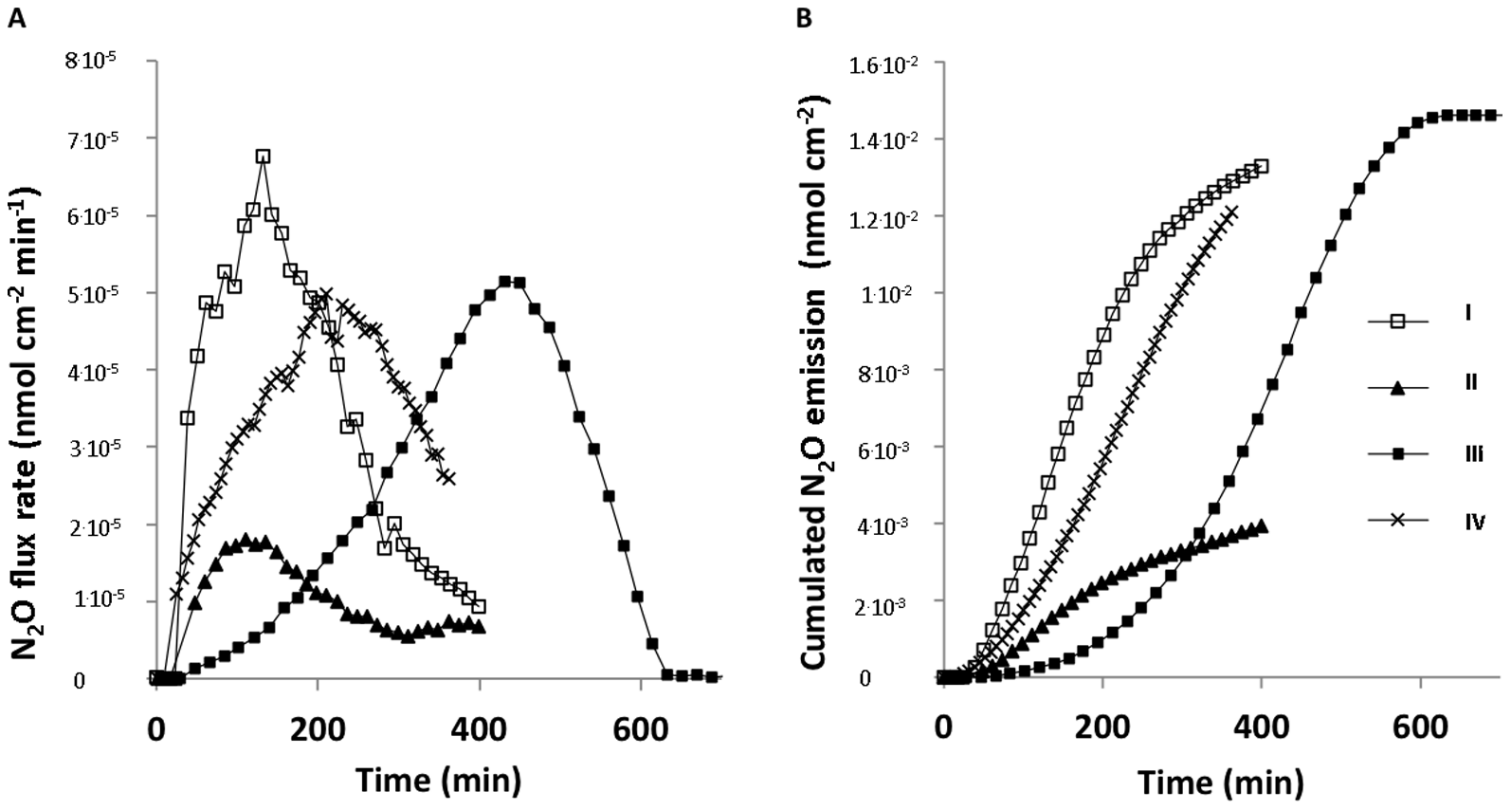
Efflux and cumulated emission of N_2_O from sputum samples. (A) Estimated N_2_O efflux rates in sputum samples from cystic fibrosis patients with chronic *P. aeruginosa* lung infection as calculated from N_2_O microprofiles (n = 4). (B) Cumulated N_2_O emission as calculated from N_2_O microprofiles (n = 4). I, II, III and IV represents 4 different sputum samples.

Measurements of the N_2_O concentration dynamics over time in particular depths of a sputum sample showed an initial build-up of N_2_O in layers below 7 mm (Fig. 5). In each layer, the slope of the net production curves was quasi-linear after ∼180 min indicating a constant production of N_2_O related to the particular layer and therefore that N_2_O originates from immobile sources such as biofilm. The production ceased about 6–7 h after start of the sample incubation, and was then followed by a net consumption of N_2_O over the following 4–5 h leading to N_2_O depletion in the sputum sample after ∼10–12 hours. In 4 sputum samples it was possible to estimate N_2_O production and consumption rates (Table 1) and N_2_O flux rates and cumulated emission (Figure 6) from measurements of such dynamic N_2_O concentration micro-gradients. A substantial initial N_2_O concentration was observed in the anaerobic zone of the remaining 4 assayed sputum samples. In these samples the N_2_O concentration decreased steadily during incubation.

**Table 1:** N_2_O production, consumption, max emission, and cumulated emission in 4 CF sputum samples.

	Net production rate	Net consumption rate	Max emission	Cumulated emission
	(nmol cm^−3^min^−1^)	(nmol cm^−3^min^−1^)	(nmol cm^−2^ min^−1^)	(nmol cm^−2^)
Median	0.47	−0.39	5.06×10^−5^	1.05×10^−2^
Range	0.40–0.70	−0.77–−0.10	1.8×10^−5^–6.78×10^−5^	3.94×10^−3^–1.46×10^−2^

## Discussion

The ability of microorganisms to exploit a wide range of electron acceptors for ATP generation by oxidative phosphorylation provides metabolic flexibility in transient environments as these organisms inhabit a variety of habitats ranging from soils, sediments to aquatic environments [19]. Even though several human pathogens, including *P. aeruginosa*, are equipped with the genetic setup for denitrification [20]–[22] including nitric oxide reductase (NOR) [22], we present the very first observations of N_2_O production in clinical material from infected human airways demonstrating pathogenic metabolism based on denitrification. These data indicate that denitrification may serve as an alternative metabolic pathway allowing *P. aeruginosa* to thrive in O_2_ depleted micro niches in the airways of CF patients. Besides our study, denitrification in humans has previously been demonstrated in human dental plaque [23] and has been related to infections of the gastrointestinal tract by the increased concentration of N_2_O in exhaled breath from patients after oral intake of NO_3_^−^ [24].

Seminal observations of O_2_ depletion and the presence of OprF porin, which is involved in NO_3_^−^ and NO_2_^−^ diffusion, in habitats of *P. aeruginosa* during chronic lung infection of CF patients provided initial evidence for anaerobic respiration by denitrification [6], [16]. To demonstrate denitrification we have included CF patients, who suffered from chronic *P. aeruginosa* infection in the endobronchial mucus as detected by routine culturing. We revealed a depletion of O_2_ in CF sputum samples, which is in accordance with the steep O_2_ gradients in endobronchial CF mucus [6] and due to O_2_ consumption by activated PMNs for generation of ROS [14]. Our O_2_ measurements in sputum confirmed the presence of O_2_ concentration gradients reaching anoxia ∼3 mm below the sputum surface.

The depletion of O_2_ for microbial respiration in infected endobronchial CF mucus has motivated the present and several other studies of anaerobic metabolism by *P. aeruginosa* based on denitrification during chronic lung infection in CF. We demonstrated N_2_O production and consumption in the sputum samples indicating the presence of active NOR and nitrous oxide reductase (N_2_OR) for the reduction of nitric oxide (NO•) and N_2_O [17]. Previously, NOR has been isolated from *P. aeruginosa* [25], the genes (*norCB*) have been sequenced [26] and functional NOR has been observed in clinical strains of *P. aeruginosa* by consumption of NO• [27].

In our study, the initial phase of N_2_O production in the sputum samples was followed by a period of net N_2_O consumption suggesting a depletion of NO• and a concomitant reduction of N_2_O to N_2_ by N_2_OR. The N_2_O consumption is in agreement with the demonstration of N_2_OR activity and the identification of the *nos* genes in *P. aeruginosa* [28] as well as the induced genes for a N_2_OR precursor in clinical isolates [29].

Our demonstration of significant N_2_O production in sputum indicates ample presence of NO_3_^−^ and NO_2_^−^ that serve as electron acceptors for the denitrification pathway. We found high levels of NO_3_^−^ and NO_2_^−^ in the sputum, which are in agreement with previous findings [30]–[32]. It has been proposed that NO_3_^−^ and NO_2_^−^ in CF sputum originates from the rapid reaction between superoxide (O_2_^-^) and NO• [15]. In this regard, we suggest the summoned activated PMNs [14] as a major source of O_2_^-^, while NO•, which is present in CF exhaled breath [33], [34], may be produced by a variety of cells in the lungs. In fact, inhalation of NO• or incubation of sputum samples with NO• resulted in elevated levels of NO_3_^−^ and NO_2_^−^ in sputum from CF patients [35]. In addition, ongoing activity of the patients nitric oxide synthases was evidenced by the increased exhaled NO• from infected CF patients following supplementation with the substrate L-arginine [36], [37].

As a consequence of our demonstration of N_2_O production, we expected a consumption of the precursors NO_3_^−^ and NO_2_^−^. Accordingly, NO_3_^−^ was depleted in the sputum after incubation for 1 day, which likely is due to the membrane-bound nitrate reductase of *P. aeruginosa* [29]. NO_3_^−^ consumption may also accompany assimilatory denitrification and ammonification resulting in the formation of ammonia (NH_4_^+^) [17], which has been detected in CF sputum [27]. However, assimilatory denitrification and ammonification does not involve production of N_2_O [17], [38], [39] and NH_4_^+^ is also produced by several human cell types [40]. The concentration of NO_2_^−^ was not changed during 1 day of incubation, but after 2 days of incubation the concentration of NO_2_^−^ in the sputum was decreased significantly. This indicates that the production of NO• from NO_2_^−^ is slower than the generation of NO_2_^−^ resulting from reduction of NO_3_^−^. Indeed, during reduction of NO_3_^−^ transient accumulation of NO_2_^−^ is known from anaerobic cultures of *P. aeruginosa* growing by denitrification [16], [41], [42].

A further verification of ongoing dissimilatory denitrification in sputum is evident from the calculated rate of N_2_O production (Fig. 6A), which easily can explain the depletion of NO_3_^−^ during incubation (Fig. 2A). The depletion of NO_3_^−^ in the sputum samples indicates that the NO_3_^−^ in sputum samples is not replaced by the reaction between O_2_^-^ and NO. This is possibly due to lack of contributions from immigrating PMNs and the epithelia as opposed to the conditions in the endobronchial mucus.

Since we calculated the rates of N_2_O production by assuming linear changes between subsequent measurements in the beginning of incubation, the estimates are likely to reflect the situation in the endobronchial mucus, where reduced NO_3_^−^ and NO_2_^−^ is continuously being replaced as indicated by the high NO_3_^−^ and NO_2_^−^ content in fresh sputum. The estimated N_2_O production, however, is calculated from the actual N_2_O content and does not include the reduction of N_2_O to N_2_. Therefore, the actual rate of denitrification may be higher than our estimates.

We found the highest concentration of N_2_O in the anoxic zone of the confined sputum samples indicating higher rate of denitrification without O_2_ as previously demonstrated [43]. Accordingly, we suggest that the low concentration of N_2_O found in the oxygenated zone is mainly due to diffusion from the active anoxic zone. Additionally, our estimate of the depth of the oxygenated zone implies that the bronchi, with diameters ranging from 0.8 to 13 mm [44], [45], allow for numerous anoxic zones in the endobronchial mucus of the lungs and confirms the *in vivo* demonstration of O_2_ depletion in the endobronchial mucus [6]. Consequently, our results propose the existence of several zones with N_2_O production in the anoxic endobronchial mucus of the lungs of CF patients with chronic *P. aeruginosa* lung infection. However, such *in vivo* production of N_2_O in CF patients still awaits direct experimental confirmation.

The involvement of denitrification enzymes as terminal oxidases that reduce nitrogen oxides in the highly branched respiratory chain of *P. aeruginosa* may enable anaerobic growth in the presence of nitrate or nitrite [19], [46]. But the engagement of denitrification in *P. aeruginosa* may also contribute to virulence as evidenced by the finding of antibodies directed against components of denitrification in CF patients with *P. aeruginosa* lung infection [16], [47] and the dependence on nitrite reductase for type III secretion [48]. In anaerobic cultures, denitrification promotes growth of *P. aeruginosa* [49], increases antibiotic tolerance of *P. aeruginosa* [50] and favors maintenance of the virulent mucoid phenotype [30].

A particular contribution to the pathogenesis of chronic lung infection in CF by NOR activity, is suggested by the induced *in vivo* gene expression in clinical isolates [29] including the highly virulent mucoid isolates [51]. In this respect, the reduction of NO• to N_2_O by active NOR may actually protect *P. aeruginosa* from the bactericidal action of NO• generated by the immune system. In fact, NOR-deficient *P. aeruginosa* is more susceptible to NO• generated by macrophages [52] and less virulent during infection of silkworm [53]. In addition, NOR activity increases the virulence of several pathogens [54]–[56].

In conclusion, this study points to the presence of anoxic microenvironments with strong spatio-temporal heterogeneity as well as a possible stratification of metabolic processes in the biofilm aggregates characteristic of chronic *P. aeruginosa* infections in the airways of CF patients. Such structural and metabolic heterogeneity may be a characteristic trait ensuring persistent infection. Indeed, spatio-temporal resolved measurements enabled the demonstrated of N_2_O production in the anaerobic zones of freshly expectorated sputum samples from CF patients with chronic *P. aeruginosa* lung infection for the first time. Analysis of the N_2_O production rates suggests ongoing generation of N_2_O in the lungs of CF patients with chronic *P. aeruginosa* infection. N_2_O production by *P. aeruginosa* in this environment is associated with anaerobic growth, which can promote increased virulence and tolerance to antibiotic, as well as contribute to evasion of the host response. The chronic infected CF lung is in many ways a black box. By using the presented approach to elucidate the essential metabolites we may now open the black box and start mapping the micro-environment of infection which may inspire new strategies for prevention and treatment of chronic lung infections in CF.

## Materials and Methods

### Sputum Samples

As defined by the “Danish Act on Research Ethics Review of Health Research Projects” Section 2 the project does not constitute a health research project and was thus initiated without approval from The Committees on Health Research Ethics in the Capital Region of Denmark. Therefore, verbal informed consent was obtained using waiver of documentation of consent. The study was carried out on 21 anonymized samples of surplus expectorated sputum from 21 CF patients and 2 PCD patients (Table 2). Chronic *P. aeruginosa* infection was defined as the presence of *P. aeruginosa* in the lower respiratory tract at each monthly culture for >6 months, or for a shorter time in the presence of increased antibody response to *P. aeruginosa* (>2 precipitating antibodies, normal: 0–1) [57].

**Table 2:** Demographic data of the patients.

	CF patients		PCD patients
	Infectious status		
	*P. aeruginosa*	*S. aureus*	*H. influenzae*
Number (male)	20 (10)	1 (1)	2 (0)
Age (years)*	39 (24–50)	17	32 (27–36)
Duration of chronic infection (years)* ^,^ **	19 (4–38)		
FEV_1_ (%)*	56 (23–96)	75	89 (70–109)
FVC (%)*	88 (46–139)	82	110 (95–125)

*Values are medians (range).

*Duration of chronic infection is only recorded for *P. aeruginosa* infections.

FEV_1_, forced expiratory volume in 1 s.

FVC, forced vital capacity.

#### Microsensor Measurements of O_2_ and N_2_O

Each of 8 different sputum samples (1–2 ml) was added to a glass vial (35×12 mm) (Schuett Biotec, Germany) and allowed to settle for about 10 min. The glass vials were positioned in a heated metal rack, kept at 37°C. Vertical O_2_-concentration profiles were recorded in the sputum with an amperometric O_2_ microsensor (OX25, Unisense A/S, Århus, Denmark) mounted in a motorized PC-controlled profiling setup (MM33 and MC-232, Unisense A/S). Subsequently, vertical N_2_O concentration profiles were recorded at defined time intervals for up to 12 hours with an amperometric N_2_O microsensor [18] (N_2_O-25, Unisense A/S) mounted in the micromanipulator.

The microsensors (tip diameter 25 µm) were connected to a picoammeter (PA2000, Unisense A/S) and positioned manually onto the upper surface of the sputum sample. Profile measurements were taken by movement of the sensor in vertical steps of 100 or 200 µm through the sputum sample. Positioning and data acquisition were controlled by dedicated software (Sensortrace Pro 2.0, Unisense A/S). The software was set to wait 3 seconds for the O_2_-microprofile and 5 seconds for the N_2_O-microprofile, before actual measurement and subsequent movement of the sensors to the next measuring depth. The interval between each cycle of profile measurements was 10 seconds.

The O_2_-microsensor was linearly calibrated by measuring the sensor signal in an alkaline sodium ascorbate solution (zero O_2_) and in air saturated free phosphate buffered saline (PBS) at experimental temperature and salinity. The O_2_ concentration in air saturated water was determined from the known temperature and salinity according to [58]. The N_2_O -microsensor was linearly calibrated according to [18] by measuring sensor signals in N_2_O free PBS at experimental temperature and salinity and in PBS with sequential addition of a known volume of N_2_O saturated PBS up to a final concentration of 100 µM N_2_O. The N_2_O concentration in saturated PBS was determined according to [59].

#### NO_3_^−^ and NO_2_^−^ quantification

The concentration of NO_3_^−^ and NO_2_^−^ in sputum was measured in 20 samples. From each sputum sample, 0.1 ml was aspired with a syringe and was immediately diluted 10x in PBS and stored at -20°C for later analysis. The remaining sample was incubated in a glass vial at 37°C for 24 h before dilution 10x in PBS and storage at −20°C. The NO_3_^−^ and NO_2_^−^ levels in the sputum were measured using the Griess colorimetric reaction (no. 780001, Cayman Chemicals, USA) according to the manufacturer's recommendations. For this, sputum samples were transferred to a 96 well microtiter plate. NO_2_^−^ concentration was estimated by addition of the Griess Reagent for 10 minutes, whereby NO_2_^−^ was converted into a purple azo-compound, which was quantitated by the optical density at 540–550 nm measured with an ELISA plate reader (Thermo Scientific Multiskan EX, Thermo Fisher Scientific Inc, BioImage, Denmark). Total NO_3_^−^ and NO_2_^−^ levels were estimated by a two-step analysis process: The first step converted NO_3_^−^ to NO_2_^−^ utilizing NO_3_^−^ reductase. After incubation for 2 hours, the next step involved the addition of the Griess Reagent, whereby NO_2_^−^ was converted into a purple azo-compound. After incubation with Griess Reagent for 10 minutes, the optical density at 540–550 nm was measured with an ELISA plate reader (Thermo Scientific Multiskan EX, Thermo Fisher Scientific Inc, BioImage, Denmark). A NO_3_^−^ standard curve was used for determination of total NO_3_^−^ and NO_2_^−^ concentration, while a NO_2_^−^ standard curve was used for determination of NO_2_^−^ alone. The concentration of NO_3_^−^ was calculated as the difference between the NO_3_^−^ concentration and the total NO_3_^−^ and NO_2_^−^ concentration.

#### Calculations of N_2_O production rates

The local N_2_O fluxes in sputum samples were calculated from the measured N_2_O concentration gradient in the uppermost oxic sputum layer. It was assumed that no production or consumption of N_2_O occurred in the presence of O_2_. The flux was calculated using a modified version of Fick's 1^st^ law of diffusion [60], where the slope of the profile in the sputum surface layer was calculated from the three uppermost measured concentrations (measurement a, b and c): 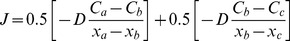
where *J* is the flux of N_2_O (nmol N_2_O cm^−2^ min^−1^), D is the molecular diffusion coefficient of N_2_O in water at 37°C (2.76×10^−5^ cm^2^ s^−1^) [61] and C is the concentration of N_2_O (µmol liter^−1^) at depth x_n_, where n = a, b or c denote 3 subsequent depths of measurement. The cumulated N_2_O emission was calculated by assuming linear changes between subsequent measurements. Net production and net consumption rates of N_2_O in particular sputum layers were calculated from the slopes of linear increase and decrease of N_2_O concentration at particular measuring depths in the sputum samples [62], [63].

#### Statistical Analyses

Statistical significance was evaluated by Wilcoxon Signed Rank Test and by Students T-test. A p value <0.05 was considered statistically significant. The tests were performed with Prism 4.0c (GraphPad Software, La Jolla, California, USA).

## References

[1] Riordan JR, Rommens JM, Kerem B, Alon N, Rozmahel R, et al (1989) Identification of the cystic fibrosis gene: cloning and characterization of complementary DNA. Science 245: 1066–1073. 2475911 10.1126/science.2475911

[2] Knowles MR, Boucher RC (2002) Mucus clearance as a primary innate defense mechanism for mammalian airways. J Clin Invest 109: 571–577. 11877463 10.1172/JCI15217 PMC150901

[3] Boucher RC (2007) Evidence for airway surface dehydration as the initiating event in CF airway disease. J Intern Med 261: 5–16. 17222164 10.1111/j.1365-2796.2006.01744.x

[4] Koch C, Høiby N (1993) Pathogenesis of cystic fibrosis. Lancet 341: 1065–1069. 7682274 10.1016/0140-6736(93)92422-p

[5] Koch C, Høiby N (2000) Diagnosis and treatment of cystic fibrosis. Respiration 67: 239–247. 10867591 10.1159/000029503

[6] Worlitzsch D, Tarran R, Ulrich M, Schwab U, Cekici A, et al (2002) Effects of reduced mucus oxygen concentration in airway *Pseudomonas* infections of cystic fibrosis patients. J Clin Invest 109: 317–325. 11827991 10.1172/JCI13870 PMC150856

[7] Bjarnsholt T, Jensen PØ, Fiandaca MJ, Pedersen J, Hansen CR, et al (2009) *Pseudomonas aeruginosa* biofilms in the respiratory tract of cystic fibrosis patients. Pediatr Pulmonol 44: 547–558. 19418571 10.1002/ppul.21011

[8] Yang L, Haagensen JA, Jelsbak L, Johansen HK, Sternberg C, et al (2008) In situ growth rates and biofilm development of *Pseudomonas aeruginosa* populations in chronic lung infections. J Bacteriol 190: 2767–2776. 18156255 10.1128/JB.01581-07 PMC2293235

[9] Pedersen SS, Moller H, Espersen F, Sorensen CH, Jensen T, et al (1992) Mucosal immunity to *Pseudomonas aeruginosa* alginate in cystic fibrosis. APMIS 100: 326–334. 1581041

[10] Bjarnsholt T, Jensen PØ, Burmolle M, Hentzer M, Haagensen JA, et al (2005) *Pseudomonas aeruginosa* tolerance to tobramycin, hydrogen peroxide and polymorphonuclear leukocytes is quorum-sensing dependent. Microbiology 151: 373–383. 15699188 10.1099/mic.0.27463-0

[11] Jensen PØ, Bjarnsholt T, Phipps R, Rasmussen TB, Calum H, et al (2007) Rapid necrotic killing of polymorphonuclear leukocytes is caused by quorum-sensing-controlled production of rhamnolipid by *Pseudomonas aeruginosa* . Microbiology 153: 1329–1338. 17464047 10.1099/mic.0.2006/003863-0

[12] van GM, Christensen LD, Alhede M, Phipps R, Jensen PØ, et al (2009) Inactivation of the rhlA gene in *Pseudomonas aeruginosa* prevents rhamnolipid production, disabling the protection against polymorphonuclear leukocytes. APMIS 117: 537–546. 19594494 10.1111/j.1600-0463.2009.02466.x PMC2997331

[13] Alhede M, Bjarnsholt T, Jensen PØ, Phipps RK, Moser C, et al (2009) *Pseudomonas aeruginosa* recognizes and responds aggressively to the presence of polymorphonuclear leukocytes. Microbiology 155: 3500–3508. 19643762 10.1099/mic.0.031443-0

[14] Kolpen M, Hansen CR, Bjarnsholt T, Moser C, Christensen LD, et al (2010) Polymorphonuclear leucocytes consume oxygen in sputum from chronic *Pseudomonas aeruginosa* pneumonia in cystic fibrosis. Thorax 65: 57–62. 19846469 10.1136/thx.2009.114512

[15] Hassett DJ, Cuppoletti J, Trapnell B, Lymar SV, Rowe JJ, et al (2002) Anaerobic metabolism and quorum sensing by *Pseudomonas aeruginosa* biofilms in chronically infected cystic fibrosis airways: rethinking antibiotic treatment strategies and drug targets. Adv Drug Deliv Rev 54: 1425–1443. 12458153 10.1016/s0169-409x(02)00152-7

[16] Yoon SS, Hennigan RF, Hilliard GM, Ochsner UA, Parvatiyar K, et al. (2002) *Pseudomonas aeruginosa* anaerobic respiration in biofilms: relationships to cystic fibrosis pathogenesis. Dev Cell 3: : 593–603. S1534580702002952 [pii]. 10.1016/s1534-5807(02)00295-2 12408810

[17] Zumft WG (1997) Cell biology and molecular basis of denitrification. Microbiol Mol Biol Rev 61: 533–616. 9409151 10.1128/mmbr.61.4.533-616.1997 PMC232623

[18] Andersen K, Kjær T, Revsbech NP (2001) An oxygen insentitive microsensor for nitrous oxide. Sensors & Actuators B: Chemical 81: 42–48.

[19] Richardson DJ (2000) Bacterial respiration: a flexible process for a changing environment. Microbiology 146 (Pt 3): 551–571. 10746759 10.1099/00221287-146-3-551

[20] Philippot L (2002) Denitrifying genes in bacterial and *Archaeal* genomes. Biochim Biophys Acta 1577: 355–376. 12359326 10.1016/s0167-4781(02)00420-7

[21] Philippot L (2005) Denitrification in pathogenic bacteria: for better or worst? Trends Microbiol 13: 191–192. 15866033 10.1016/j.tim.2005.03.001

[22] Zumft WG (2005) Nitric oxide reductases of prokaryotes with emphasis on the respiratory, heme-copper oxidase type. J Inorg Biochem 99: 194–215. 15598502 10.1016/j.jinorgbio.2004.09.024

[23] Schreiber F, Stief P, Gieseke A, Heisterkamp IM, Verstraete W, et al (2010) Denitrification in human dental plaque. BMC Biol 8: 24. 20307293 10.1186/1741-7007-8-24 PMC2859859

[24] Mitsui T, Kondo T (2004) Increased breath nitrous oxide after ingesting nitrate in patients with atrophic gastritis and partial gastrectomy. Clin Chim Acta 345: 129–133. 15193987 10.1016/j.cccn.2004.03.011

[25] Hino T, Matsumoto Y, Nagano S, Sugimoto H, Fukumori Y, et al (2010) Structural basis of biological N_2_O generation by bacterial nitric oxide reductase. Science 330: 1666–1670. 21109633 10.1126/science.1195591

[26] Arai H, Igarashi Y, Kodama T (1995) The structural genes for nitric oxide reductase from *Pseudomonas aeruginosa* . Biochim Biophys Acta 1261: 279–284. 7711073 10.1016/0167-4781(95)00018-c

[27] Gaston B, Ratjen F, Vaughan JW, Malhotra NR, Canady RG, et al (2002) Nitrogen redox balance in the cystic fibrosis airway: effects of antipseudomonal therapy. Am J Respir Crit Care Med 165: 387–390. 11818326 10.1164/ajrccm.165.3.2106006

[28] SooHoo CK, Hollocher TC (1991) Purification and characterization of nitrous oxide reductase from *Pseudomonas aeruginosa* strain P2. J Biol Chem 266: 2203–2209. 1899237

[29] Son MS, Matthews WJ Jr, Kang Y, Nguyen DT, Hoang TT (2007) In vivo evidence of *Pseudomonas aeruginosa* nutrient acquisition and pathogenesis in the lungs of cystic fibrosis patients. Infect Immun 75: 5313–5324. 17724070 10.1128/IAI.01807-06 PMC2168270

[30] Hassett DJ (1996) Anaerobic production of alginate by *Pseudomonas aeruginosa*: alginate restricts diffusion of oxygen. J Bacteriol 178: 7322–7325. 8955420 10.1128/jb.178.24.7322-7325.1996 PMC178651

[31] Jones KL, Hegab AH, Hillman BC, Simpson KL, Jinkins PA, et al (2000) Elevation of nitrotyrosine and nitrate concentrations in cystic fibrosis sputum. Pediatr Pulmonol 30: 79–85. 10922128 10.1002/1099-0496(200008)30:2<79::aid-ppul1>3.0.co;2-1

[32] Palmer KL, Brown SA, Whiteley M (2007) Membrane-bound nitrate reductase is required for anaerobic growth in cystic fibrosis sputum. J Bacteriol 189: 4449–4455. 17400735 10.1128/JB.00162-07 PMC1913347

[33] Grasemann H, Michler E, Wallot M, Ratjen F (1997) Decreased concentration of exhaled nitric oxide (NO) in patients with cystic fibrosis. Pediatr Pulmonol 24: 173–177. 9330413 10.1002/(sici)1099-0496(199709)24:3<173::aid-ppul2>3.0.co;2-o

[34] Linnane SJ, Keatings VM, Costello CM, Moynihan JB, O'Connor CM, et al (1998) Total sputum nitrate plus nitrite is raised during acute pulmonary infection in cystic fibrosis. Am J Respir Crit Care Med 158: 207–212. 9655731 10.1164/ajrccm.158.1.9707096

[35] Ratjen F, Gartig S, Wiesemann HG, Grasemann H (1999) Effect of inhaled nitric oxide on pulmonary function in cystic fibrosis. Respir Med 93: 579–583. 10542992 10.1016/s0954-6111(99)90158-0

[36] Grasemann H, Grasemann C, Kurtz F, Tietze-Schillings G, Vester U, et al (2005) Oral L-arginine supplementation in cystic fibrosis patients: a placebo-controlled study. Eur Respir J 25: 62–68. 15640324 10.1183/09031936.04.00086104

[37] Grasemann H, Tullis E, Ratjen F (2013) A randomized controlled trial of inhaled l-Arginine in patients with cystic fibrosis. J Cyst Fibros. Available: http://dx.doi.org/10.1016/j.jcf.2012.12.008. 10.1016/j.jcf.2012.12.008 23333044

[38] Einsle O, Messerschmidt A, Stach P, Bourenkov GP, Bartunik HD, et al (1999) Structure of cytochrome c nitrite reductase. Nature 400: 476–480. 10440380 10.1038/22802

[39] Einsle O, Messerschmidt A, Huber R, Kroneck PMH, Neese F (2002) Mechanism of the six-electron reduction of nitrite to ammonia by cytochrome c nitrite reductase. J Am Chem Soc 124: ; 11737–11745. 10.1021/ja0206487 12296741

[40] Planelles G (2007) Ammonium homeostasis and human Rhesus glycoproteins. Nephron Physiol 105: ; 11–17. 10.1159/000096979 17106214

[41] Williams DR, Rowe JJ, Romero P, Eagon RG (1978) Denitrifying *Pseudomonas aeruginosa*: some parameters of growth and active transport. Appl Environ Microbiol 36: 257–263. 100056 10.1128/aem.36.2.257-263.1978 PMC291211

[42] Hoffman LR, Richardson AR, Houston LS, Kulasekara HD, Martens-Habbena W, et al (2010) Nutrient availability as a mechanism for selection of antibiotic tolerant *Pseudomonas aeruginosa* within the CF airway. PLoS Pathog 6: e1000712. 20072604 10.1371/journal.ppat.1000712 PMC2795201

[43] Thomas KL, Lloyd D, Boddy L (1994) Effects of oxygen, pH and nitrate concentration on denitrification by *Pseudomonas* species. FEMS Microbiol Lett 118: 181–186. 8013877 10.1111/j.1574-6968.1994.tb06823.x

[44] Seneterre E, Paganin F, Bruel JM, Michel FB, Bousquet J (1994) Measurement of the internal size of bronchi using high resolution computed tomography (HRCT). Eur Respir J 7: 596–600. 8013616 10.1183/09031936.94.07030596

[45] Hampton T, Armstrong S, Russell WJ (2000) Estimating the diameter of the left main bronchus. Anaesth Intensive Care 28: 540–542. 11094671 10.1177/0310057X0002800510

[46] Arai H (2011) Regulation and Function of Versatile Aerobic and Anaerobic Respiratory Metabolism in *Pseudomonas aeruginosa* . Front Microbiol 2: 103. 21833336 10.3389/fmicb.2011.00103 PMC3153056

[47] Beckmann C, Brittnacher M, Ernst R, Mayer-Hamblett N, Miller SI, et al (2005) Use of phage display to identify potential *Pseudomonas aeruginosa* gene products relevant to early cystic fibrosis airway infections. Infect Immun 73: 444–452. 15618183 10.1128/IAI.73.1.444-452.2005 PMC538986

[48] Van Alst NE, Wellington M, Clark VL, Haidaris CG, Iglewski BH (2009) Nitrite reductase NirS is required for type III secretion system expression and virulence in the human monocyte cell line THP-1 by *Pseudomonas aeruginosa* . Infect Immun 77: 4446–4454. 19651860 10.1128/IAI.00822-09 PMC2747934

[49] Williams DR, Rowe JJ, Romero P, Eagon RG (1978) Denitrifying *Pseudomonas aeruginosa*: some parameters of growth and active transport. Appl Environ Microbiol 36: 257–263. 100056 10.1128/aem.36.2.257-263.1978 PMC291211

[50] Borriello G, Werner E, Roe F, Kim AM, Ehrlich GD, et al (2004) Oxygen limitation contributes to antibiotic tolerance of *Pseudomonas aeruginosa* in biofilms. Antimicrob Agents Chemother 48: 2659–2664. 15215123 10.1128/AAC.48.7.2659-2664.2004 PMC434183

[51] Lee B, Schjerling CK, Kirkby N, Hoffmann N, Borup R, et al (2011) Mucoid *Pseudomonas aeruginosa* isolates maintain the biofilm formation capacity and the gene expression profiles during the chronic lung infection of CF patients. APMIS 119: 263–274. 21492226 10.1111/j.1600-0463.2011.02726.x

[52] Kakishima K, Shiratsuchi A, Taoka A, Nakanishi Y, Fukumori Y (2007) Participation of nitric oxide reductase in survival of *Pseudomonas aeruginosa* in LPS-activated macrophages. Biochem Biophys Res Commun 355: 587–591. 17307144 10.1016/j.bbrc.2007.02.017

[53] Arai H, Iiyama K (2013) Role of nitric oxide-detoxifying enzymes in the virulence of *Pseudomonas aeruginosa* against the silkworm, *Bombyx mori* Biosci Biotechnol Biochem 77: : 198–200. DN/JST.JSTAGE/bbb/120656 [pii]. 10.1271/bbb.120656 23291757

[54] Shimizu T, Tsutsuki H, Matsumoto A, Nakaya H, Noda M (2012) The nitric oxide reductase of enterohaemorrhagic *Escherichia coli* plays an important role for the survival within macrophages. Mol Microbiol 85: 492–512. 22716767 10.1111/j.1365-2958.2012.08122.x

[55] Stevanin TM, Moir JW, Read RC (2005) Nitric oxide detoxification systems enhance survival of *Neisseria meningitidis* in human macrophages and in nasopharyngeal mucosa. Infect Immun 73: 3322–3329. 15908358 10.1128/IAI.73.6.3322-3329.2005 PMC1111883

[56] Loisel-Meyer S, Jimenez de Bagues MP, Basseres E, Dornand J, Kohler S, et al (2006) Requirement of norD for *Brucella suis* virulence in a murine model of in vitro and in vivo infection. Infect Immun 74: 1973–1976. 16495577 10.1128/IAI.74.3.1973-1976.2006 PMC1418625

[57] Høiby N (2000) Microbiology of cystic fibrosis. In: Hodson ME GD, editors. Cystic fibrosis. London, UK: Arnold. pp. 83–107.

[58] Gundersen JK, Glud RN, Ramsing NB (1998) Predicting the signal of O_2_ microsensors from physical dimensions, temperature, salinity, and O_2_ concentration. Limnol Oceanogr 43: 1932–1937.

[59] Weiss RF, Price BA (1980) Nitrous oxide solubility in water and seawater. Marine Chemistry 8: 347–359.

[60] de Beer D, Stoodley P (2006) Microbial biofilms. In: Dworkin M, Falkow S, Rosenberg E, Schleifer KH, Stackebrandt E, editors. The Prokaryotes. New York: Springer Science. pp. 904–937.

[61] Broecker WS, Peng TH (1974) Gas-exchange rates between air and sea. Tellus 26: 21–35.

[62] Markfoged R, Nielsen LP, Nyord T, Ottosen LDM, Revsbech NP (2013) Transient N_2_O accumulation and emission caused by O_2_ depletion in soil after liquid manure injection. European journal of soil science 62: 541–550.

[63] Liengaard L, Nielsen LP, Revsbech NP, Priemé A, Elberling B, et al (2013) Extreme emission of N_2_O from tropical wetland soil (Pantanal, South America). Frontiers in Microbiology 3: 433 doi: 10.3389/fmicb.2012.00433 23293634 10.3389/fmicb.2012.00433 PMC3537118

